# Impact of drug reconciliation at discharge and communication between hospital and community pharmacists on drug-related problems: study protocol for a randomized controlled trial

**DOI:** 10.1186/1745-6215-15-260

**Published:** 2014-06-30

**Authors:** Xavier Pourrat, Clarisse Roux, Brigitte Bouzige, Valérie Garnier, Armelle Develay, Benoit Allenet, Martial Fraysse, Jean-Michel Halimi, Jacqueline Grassin, Bruno Giraudeau

**Affiliations:** 1 Pharmacy Department, Pharm D, Tours University Hospital, 2 boulevard Tonnelle, 37044 Tours cedex 09, France; 2 Pharmacy Department, Pharm D, Nîmes University Hospital, 4 Rue du Professeur Robert Debré, 30029 Nîmes, France; 3 Pharm D, Pharmacie Bouzige, 32 Rue Pont, 30110 Les Salles du Gardon, France; 4 Pharm D, Pharmacien, Pharmacie Garnier, 1 Chemin des Prés, 30840 Meynes, France; 5 Pharmacy Department, Pharm D, CHU de Grenoble, Boulevard de la Chantourne, 38700 La Tronche, France; 6 Pharm D, 52 Rue du Commandant Jean Duhail, 94120 Fontenay-sous-Bois, France; 7 Nephrology Department, Tours University Hospital, 2 boulevard Tonnelle, 37044 Tours cedex 09, France; 8 INSERM CIC 1415, CHRU de Tours, Hôpital Bretonneau, 2 Boulevard Tonnellé, 37044 Tours Cedex 09, France; 9 ThEMAS TIMC-IMAG (UMR CNRS 5525), School of Medicine and Pharmacy, J Fourier University, Grenoble, 621 Avenue Centrale, 38041 Saint-Martin-d'Hères, France; 10 Therapeutic Department, School of Medicine, François Rabelais University, 10 Boulevard Tonnellé, 37000 Tours, France; 11 INSERM CIC 1415, François Rabelais University, 10 Boulevard Tonnellé, 37000 Tours, France

**Keywords:** Community pharmacist, hospital pharmacist, drug-related problem, communication, cluster randomized crossover trial, hospital discharge, drug reconciliation

## Abstract

**Background:**

Patients are at risk of drug-related problems (DRPs) at transition points during hospitalization. The community pharmacist (CP) is often the first healthcare professional patients visit after discharge. CPs lack sufficient information about the patient and so they may be unable to identify problems in medications, which may lead to dispensing the wrong drugs or dosage, and/or giving wrong information. We aim to assess the impact of a complex intervention comprising of medication reconciliation performed at discharge by a hospital pharmacist (HP) with communication between the HP and CP on DRPs during the seven days following discharge.

**Methods/Design:**

The study is a cluster randomized crossover trial involving 46 care units (each unit corresponding to a cluster) in 22 French hospitals during two consecutive 14-day periods, randomly assigned as ‘experimental’ or ‘control’ (usual care) periods. We will recruit patients older than 18 years of age and visiting the same CP for at least three months. We will exclude patients with a hospital length of stay of more than 21 days, who do not return home or those in palliative care. During the experimental period, the HP will perform a medications reconciliation that will be communicated to the patient. The HP will inform the patient’s CP about the patient’s drug therapy (modification in home medication, acute drugs prescribed, nonprescription treatments, and/or lab results). The primary outcome will be a composite outcome of any kind of drug misuse during the seven days following discharge assessed at day seven (±2) post-discharge by a pharmacist in charge of the study who will contact both patients and CPs by phone. The secondary outcome will be unplanned hospitalizations assessed by phone contact at day 35 (±5) after discharge. We plan to recruit 1,176 patients.

**Discussion:**

This study will assess the impact of a reconciliation of medications performed at patient discharge followed by communication between the HP and the patient’s CP. It will allow for identifying the type of patients in France for which the intervention is most relevant.

**Trial registration:**

This study was registered with ClinicalTrials.gov (number: NCT02006797) on 5 December 2013.

## Background

Drug-related problems (DRPs) are defined as an ‘event or circumstance involving drug therapy that actually or potentially interferes with desired health outcomes’ [1]. These problems are the cause of about 11% of the iatrogenic problems and could be avoided.

In France, the national survey of all serious adverse events associated with care (ENEIS) showed that 1.3% of hospitalizations (between 100,000 and 120,000 a year) are due to a serious drug-related iatrogenic event and are therefore avoidable [2]. Furthermore, more than half occur after a new prescription at admission or during discharge. In fact, the French health authority, through an accreditation procedure, requires hospitals to ensure treatment continuity from admission to discharge [3].

An estimated 7 to 30% of patients present a DRP at hospital admission [4]. A reconciliation of medications supported by efficient communication between the hospital staff and community pharmacists (CPs), in addition to a standard patient interview and a general practitioner’s examination of prescriptions, was found to be effective in identifying medication discrepancies for inpatients [5,6]. Approximately 25 to 87% of patients experience DRPs after hospital discharge [7-14]. Drug reconciliation before discharge was also found to be effective and could decrease DRPs by 50% when performed by a medical and/or pharmaceutical team; pharmaceutical teams were more effective in this process than medical or nursing teams [15-18].

Medication reconciliation is defined as the formal process of checking the complete, accurate list of a patient’s previous medication and comparing it with the prescriptions after a transition of care (on admission, after transfer to another medical unit, and at discharge) [19]. The process has been recommended since 2005 by the Joint Commission on Accreditation to prevent errors. Countries such as Germany, the Netherlands, and France are involved in the World Health Organization (WHO) High 5’s procedure and particularly in medication reconciliation at admission [20,21]. Discrepancies between hospital treatment and home medication must be discussed with the prescriber and modifications made if necessary [19]. In fact, non-intentional discrepancies (NIDs) or intentional discrepancies (IDs) may be observed. An ID is a voluntary change in the patient’s medication (unnecessary drugs, route or dose change, or conformation to the hospital formulary). NIDs (wrong route or dose, missing treatment, or added drug) are considered medication errors. Among NIDs, 40 to 59% are potential causes of adverse events and 33% actually lead to adverse events [19].

Several experiments have been conducted in North America or Europe to increase the quality of information at discharge, considering that well-informed patients and/or caregivers can manage the drug treatment on their own. However, few studies have focused on the role of the CP at discharge, both in the reconciliation process and/or the information needed to reduce DRPs (such as the medical discharge letter) [22,23].

The primary objective of this study is to investigate a hospital pharmacist (HP) performing a reconciliation of medications with the patient at discharge, followed by communication between the HP and the CP, and their impact on the incidence of DRPs in patients during the seven days after discharge. The potential harmfulness of DRPs will be appraised by an expert committee. Secondary objectives are patient satisfaction and subgroup analyses.

## Methods/Design

### Design

This study will be a cluster randomized crossover controlled trial. The clusters will be hospital units, each involved during two consecutive 14-day periods (one when the assessed intervention will apply and the other as the control period). For each unit, the order of the two periods will be randomized (intervention followed by control, or vice versa).

We planned the study as a cluster trial because of methodological issues. Indeed, randomization of patients would have implied fully informing them of the study, thus increasing the risk of group contamination. Conversely, randomizing clusters allows for patients to receive partial information; patients included in the usual care group will be unaware that some patients will have a reconciliation procedure performed at discharge. Therefore, we planned the study as a crossover trial because: 1) the risk of cross-contamination is minimal (residual effect on a short period is null); 2) even if a residual effect exists, it is a superiority test that would lead to minimizing the intervention effect; 3) the crossover trial allows for increasing the statistical power and thereby balancing the power lost with the cluster design [24]; and 4) a crossover design offers better balance between groups, which is of importance in our study due to the high heterogeneity in units.

### Setting and participants

In total, 22 French HPs working in hospitals all over the French country have agreed to participate. Each HP selected two units (one surgical, one medical) in their hospital. Units for which a pharmaceutical reconciliation procedure at admission or discharge was already in place were not eligible. The medical heads of the units also agreed to participate. We will exclude patients with a length of stay of more than 21 days (as there can be too many therapeutic modifications during the stay), who do not return home, who are in palliative care and/or near the end of life, and who are not able to understand the topic.

CPs working in the area around the hospital will participate by the inclusion of their patients in the study. They will be informed of the study in three ways: a professional journal supported by the pharmacist unions, a professional journal supported by the national council of the order of pharmacists, and a letter sent by the study scientific committee distributed by wholesale drug distributors.

### Outcomes

The primary outcome is a composite one comprising of any problem or dysfunction observed during the seven days after discharge. More specifically, we will consider any of the following problems: 1) the drug is not the correct one (name, form, route, or dose); 2) the patient does not take what was prescribed and/or takes treatments that should have been stopped; and 3) the patient could not obtain the medication when visiting the pharmacy, which causes a gap in the continuity and duration of therapy.

The primary outcome will be assessed at day seven (±2) after discharge, by a pharmacist specifically recruited for the study. This pharmacist will contact both patients and CPs by phone to assess the possible incidence of a DRP after discharge.

For each DRP, an expert committee composed of physicians (one nephrologist, one cardiologist, one gastroenterologist, and a clinical pharmacist) will assess the potential medical impact of the DRP in terms of its severity (from 0: no problem, to 3: life-threatening) using Bayliff’s scale [25]. The severity of the identified DRP will be one of the secondary outcomes.

Other secondary outcomes will be: 1) the number of non-planned hospitalizations during the 35 days after discharge (assessed by phone by the pharmacist recruited for the study); and 2) patients and CP satisfaction (assessed at day seven with a four-item Likert scale by phone). We will also assess the time taken by the HP to perform the intervention (medication reconciliation and communication to CP) and the proportion of drug prescriptions modified by the HP at discharge. DRP will be studied in following five subgroups: 1/less or over or equal to 75-years-old, 2/less or over or equal to 4 drugs prescribed 3/expensive or not-expensive medication 4/surgery or medical-unit and 5/planned or non-planned hospitalization.

### Intervention

**Figure 1:**
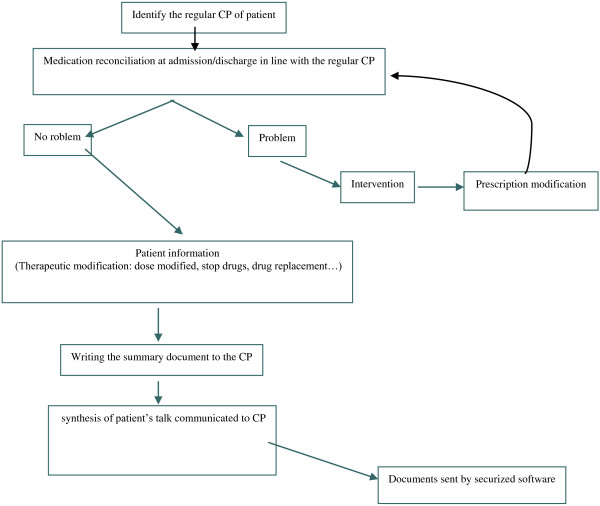
**REPHVIM study - flow of the intervention.** CP, community pharmacists.

The flow of the intervention is outlined in Figure 1. In the experimental group, the HP will be in charge of the discharge reconciliation and its communication to the CP. To standardize this nonpharmacological intervention over the different hospitals [26], HPs will receive training in the reconciliation procedure by an experienced clinical pharmacist who is accredited by the French Society of Clinical Pharmacy (SFPC).

**Figure 2:**
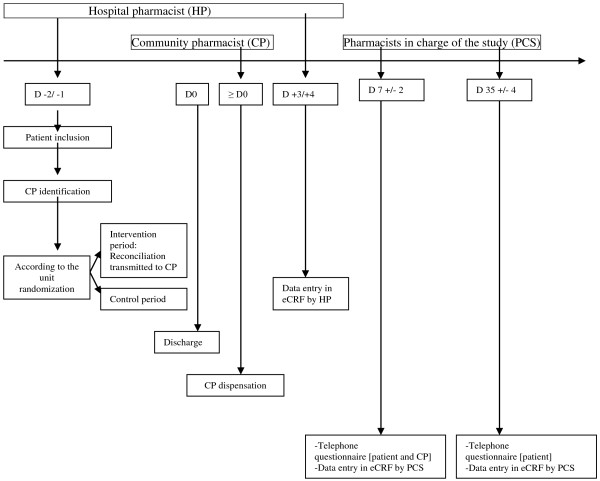
REPHVIM Study - synopsis of the study.

The intervention consists of: 1) identifying the patient’s regular CP, 2) completing a short document including the reason for hospitalization, therapeutic modifications in terms of the home medication, and lab results necessary to understand and/or accept the prescription (estimated glomerular filtration rate, Na and K levels, coagulation results, and so on), 3) controlling the discharge prescription and, if needed, discussing it with the physician and recording it on the SFPC card [27], 4) explaining the treatment to the patient and the modifications made, 5) phoning the CP to explain the patient’s inclusion in the study, the discharge time, and the modifications in treatment, and 6) sending the CP the prescription sheet via a secure email before patient discharge. The CP will then receive visits from the patient or caregiver as usual. For the control group, pharmaceutical care will be performed as usual, with no reconciliation procedure at discharge and no contact with the CP. REPHVIM study is outlined in Figure 2.

### Blinding

Because of the nature of the assessed intervention, blinding will not be possible for patients or care providers (HPs and CPs). Also, we did not include a blinded outcome assessor because blinding could be easily broken when contacting patients and CPs. Therefore, the present study is fully open, without reliable blinding. The only blinded outcome is a secondary one; the expert committee in charge of assessing the potential medical impact of the DRP will be blinded.

### Sample size calculation

This study is a superiority trial with a binary decision criterion. We hypothesized a DRP rate of 45% in the experimental group as compared to 60% in the control group [7]. Considering a 90% power and a 5% two-sided alpha level, we would need 235 patients per group with a trial of two parallel, individually randomized groups. We used a sample size calculation software (nQuery Advisor -2005- Version 6.0 Los Angeles, CA. Copyright © 2005 by Janet D. Elashoff). In addition, because the trial is a cluster randomized crossover trial, we assessed both intraclass and interclass correlations [24,28]. We therefore applied an inflation factor equal to:

(1) $$1\;+\;\left(P\;–1\right)\rho -\mathrm{P\eta}$$

where *P* is the number of patients in a cluster, *ρ* is the intraclass correlation coefficient and *η* is the interclass correlation coefficient. We considered a high value for the intraclass correlation coefficient because the outcome is a process, and because of the incidence of about 50% [29]. Naturally, *η* is expected to be lower than *ρ*, and we chose the value of *η* to be half that of *ρ* as advised [24]. We will use a value of 0.2 for *ρ* and 0.1 for *η*.

Because we identified 46 units, we planned to recruit 10.2 patients in each unit for each period. We plan to perform a sensitivity analysis excluding patients without data at day seven after discharge, and so we fixed the number of patients to be recruited in each period to 14. In the end, we expect to recruit 14 patients in each of the 46 units in each period, for 1,176 patients.

### Statistical analysis

The primary analysis will be conducted within the framework of a hierarchical model, inspired by the work of Turner *et al*. [30]. Under this model, two levels of correlation are considered: intra-cluster correlation and cross-correlation period. Secondary outcomes will also be analyzed with hierarchical models. Subgroup analyzes will consider patient characteristics (age and pathology), drugs classes, nature of the unit (surgery or medical), number of home treatments, day of discharge (weekday or weekend), whether it is an expensive drug (more than 150€ per unit) is prescribed, and whether some medications require temperature control.

### Funding source and regulatory aspects

This study is sponsored by the French Ministry of Health (PREPS 2012 number 12-10-0054). The CHRU of Tours is the promoter and is in charge of all the administrative measures. The local ethics committee (CPP TOURS - Region Centre - Ouest 1) approved the study for all centers. Indeed, French legislation requires just one ethic committee’s approval for all centers. We were asked by the CPP TOURS - Region Centre - Ouest 1 to provide a signed commitment from all the heads of the involved care units. In Table 1 we report the list of those units. Also as authorized by French law, the requirement for patient written consent was not necessary and only information about the study was to be given to patient [31]. In fact the patient can always refuse to participate. The French committees for data handling (CCTIRS and CNIL) approved the study. This trial was registered with ClinicalTrials.gov (number NCT02006797 on 5 December 2013, Relation between hospital and community pharmacists and drug-related problems (REPHVIM)).

**Table 1:** Centers and names of the units which agreed to participate to the study

**Hospital**	**Unit 1**	**Unit 2**	**Unit 3**	**Unit 4**
CH ALES	Medical	Abdominal surgery		
CHU ANGERS	Infectious diseases	Abdominal surgery 2	Abdominal surgery 1	
CH BETHUNE	Nephrology & rheumatology	Abdominal surgery		
CH BLOIS	Cardiology			
CHU BREST	Gastro-enterology	Urology		
CHU CLERMONT-FERRAND	Medical	Abdominal surgery	Gastro-enterology	
CH COLMAR	Medical	Orthopedic		
CH COMPIEGNE	Cardiology	Orthopedic		
CHU GRENOBLE	Geriatric			
CH LE HAVRE	Rheumatology	Abdominal surgery		
AP-HM (MARSEILLE)	Medical	Abdominal surgery		
CH METZ	Medical	Abdominal surgery		
CH LE MANS	Rheumatology	Abdominal surgery		
CH NEVERS	Medical	Abdominal surgery		
CHU NICE	Gastro-enterology	Abdominal surgery		
CHU NIMES	Cardiology	Urology		
CHU POITIERS	Cardiology	Orthopedic	Neurology	Vascular surgery
CHRU STRASBOURG	Oncology	Abdominal surgery		
CHU TOULOUSE	Rheumatology	Vascular surgery		
HIA BEGIN	Infectious diseases	Abdominal surgery		
CHU TOURS	Cardiology	Vascular surgery		
CHU REIMS	Unit 1	Unit 2		

### Dissemination

The scientific committee will be in charge of the publications to report the results of the present study. Reports will follow the CONSORT statement and its extensions (for both cluster randomized trials and nonpharmacological interventions), as well as the TIDER checklist.

## Discussion

This study will investigate the effect of HP reconciling medications with the patient at discharge and communicating with the patient’s CP on preventing DRPs. The subgroups study will evaluate the patients for which the process would be efficient. The French Society of Clinical Pharmacy is expected to write recommendations and promote communication between HPs and CPs. We expect a decrease in DRPs from 60 to 45%.

The success of the study depends on the ability of the HP to enroll patients in the study. The units have been chosen for their possibility of having more than one discharge a day. Most are surgery units (orthopedic, urology, or general surgery) and medical units (nephrology, gastroenterology, internal medicine, and so on) with a large number of beds (more than 20) and a mean length of stay from 5 to 11 days. Patients in the intervention group need to spend only a few minutes of discussion with the HP. This intervention will need to be performed as soon as possible once discharge is planned. Indeed, at discharge, patients are usually stressed with transport and other administrative issues, so they may be less receptive to discuss their treatment.

To optimize and standardize the intervention assessed, the scientific committee has established a training program for HPs. Also, quality documents have been written to ensure performance of the same intervention. Outcome assessments will be performed centrally by two pharmacists (pharmacists in charge of the study) specially recruited for the study, which will favor homogeneity in the phone interviews.

## Trial status

At this time, 44 units have been recruited since 21 January 2014 and 340 patients have been included.

## Abbreviations

CP: Community pharmacist; CH: Hospital (Centre Hospitalier); CHU: University hospital (Centre Hospitalier Universitaire) DRP, Drug-related problem; eDFG: Estimated Glomerular Filtration Rate; ENEIS: National Study on Iatrogenic Event Avoidable; GP: General practitioner; HP: Hospital pharmacist; ID: Intentional discrepancy; NID: Non intentional discrepancy; PCS: Pharmacist in charge of the study; PREPS: Research program on the performance of care system; SFPC: French society of clinical pharmacy.

## Competing interests

The authors declare that they have no competing interests.

## Authors’ contributions

XP: writing the protocol, submitting protocol to DGOS, acquiring the funds, enrolling the hospitals, link with SFPC, planning the study, responsible for the scientific committee. CR: test the eCRF, check the procedure for reconciliation, checking the study process. BB: co-writing the protocol, member of the scientific committee, link with the CP union. VG: co-writing the protocol, member of the scientific committee, link with the CP union. AD: co-writing the protocol, member of the scientific committee, link with SFPC. BA: co-writing the protocol, member of the scientific committee, test the eCRF. MF: co-writing the protocol, member of the scientific committee, link with the National Council of the Order of Pharmacists. JMH: co-writing the protocol, member of the scientific committee, responsible for medical experts committee. JG: co-writing and correcting the protocol, member of the scientific committee, past vice-president of SFPC. BG: writing and correcting the protocol, responsible for methodology, head of the clinical research department (responsible for clinical research associate, teams in charge of eCRF and data manager). All authors read and approved the final manuscript.
